# Advanced Consumptives

**Published:** 1920-08-07

**Authors:** 


### Advanced Consumptives.

Progress is reported with the scheme for providing a combined residential institution for advanced cases of consumption to serve the Boroughs of Lewisham, Deptford, Woolwich, and Greenwich, all four boroughs now being agreed upon the necessity for such provision, whilst the Ministry of Health and the London County Council have both expressed sympathy with the proposal. The great point in favour of the idea is that the patients will be away from their crowded homes and yet within reach of their friends. The possibilities of infection will thereby be considerably diminished for persons who now habitually come in contact with the patients. The Greenwich Medical Officer of Health has ascertained that besides being sympathetic, the Ministry of Health, the London County Council, and the London Insurance Committee undertake to subsidise, to the fullest extent of the capacity contemplated by the proposed institution, at the rate of £2 10s. per bed per week. During the negotiations with the Ministry of Health and the London County Council the scope of the proposed institution was materially widened, it now being suggested that some of the beds should be reserved for patients attending the tuberculosis dispensaries as observation beds, some for patients awaiting removal to ordinary sanatoria, and some for emergency cases. The scheme involves an annual expenditure of £4,158.

